# Differences in resting-state brain networks and gray matter between APOE ε2 and APOE ε4 carriers in non-dementia elderly

**DOI:** 10.3389/fpsyt.2023.1197987

**Published:** 2023-08-10

**Authors:** Zhiyuan Wang, Jing Pang, Ruizhi Zhou, Jianjiao Qi, Xianglong Shi, Bin Han, Xu Man, Qingqing Wang, Jinping Sun

**Affiliations:** ^1^Institute of Integrative Medicine, The Affiliated Hospital of Qingdao University, Qingdao, China; ^2^Department of Radiology, The Affiliated Hospital of Qingdao University, Qingdao, China; ^3^Department of Emergency Medicine, The Affiliated Hospital of Qingdao University, Qingdao, China; ^4^Department of Neurology, The Affiliated Hospital of Qingdao University, Qingdao, China

**Keywords:** resting-state functional magnetic resonance imaging, APOE ε2, APOE ε4, independent component analysis, voxel-based morphometry, non-dementia elderly

## Abstract

**Background:**

Apolipoprotein E (APOE) ε2 and APOE ε4 are the most distinct alleles among the three APOE alleles, both structurally and functionally. However, differences in cognition, brain function, and brain structure between the two alleles have not been comprehensively reported in the literature, especially in non-demented elderly individuals.

**Methods:**

A neuropsychological test battery was used to evaluate the differences in cognitive performance in five cognitive domains. Independent component analysis (ICA) and voxel-based morphometry (VBM) were used separately to analyze resting-state functional magnetic resonance imaging (rs-fMRI) data and the structure MRI data between the two groups. Finally, correlations between differential brain regions and neuropsychological tests were calculated.

**Results:**

APOE ε2 carriers had better cognitive performance in general cognitive, memory, attention, and executive function than APOE ε4 carriers (all *p* < 0.05). In ICA analyses of rs-fMRI data, the difference in the resting-state functional connectivity (rsFC) between two groups is shown in 7 brain networks. In addition, VBM analyses of the T1-weighted image revealed that APOE ε2 carriers had a larger thalamus and right postcentral gyrus volume and a smaller bilateral putamen volume than APOE ε4 carriers. Finally, differences in brain function and structure may be the reason that APOE ε2 carriers are better than APOE ε4 carriers in cognitive performance.

**Conclusion:**

These findings suggest that there are significant differences in brain function and structure between APOE ε2 carriers and APOE ε4 carriers, and these significant differences are closely related to their cognitive performance.

## 1. Introduction

The Apolipoprotein E (APOE) gene is an allele known to be closely associated with sporadic Alzheimer’s disease (AD) (1–3), it was an isoform composed of three alleles ε2, ε3, and ε4, and is located on chromosome 19q13.32 (4). APOE ε4 in the 3 alleles is the primary risk variant for AD and studies have shown that mice are replaced by human APOE ε4 present serious brain atrophy and neuroinflammation than mice are replaced by human APOE ε2 and ε3 (5, 6). APOE ε2 has been considered as the neuroprotective and longevity gene, which reduces the risk of AD in comparison to APOE ε3 and ε4 (7, 8). A study consisting of over 5,000 participants found that the likelihood of APOE ε2 homozygotes is significantly lower in individuals with AD compared to those with other APOE alleles (9).

Studies have shown that APOE ɛ3 is not only the most common but also has a slight effect on the carrier’s cognitive function compared to other alleles (10). Postmortem studies on patients with AD revealed that APOE ε4 accelerated the accumulation of Aβ and plaque deposition in the brain (11, 12), while APOE ε2 decreased the Aβ and plaque deposition (13). Based on the findings of these studies, APOE ε2 and APOE ε4 may affect the cognitive function of carriers via different regulations of the same pathological process (7), thus, a direct comparison of the difference in the brain of carriers between these two genotypes may be more helpful in advancing our understanding about AD.

Since the discovery by Biswal et al. (14) regarding synchronization patterns of low-frequency oscillatory signals in the sensorimotor cortex, it has opened the door for resting-state fMRI (15–18). Using rs-fMRI, Chen et al. (19) reported decreased local rsFC on medial temporal areas in APOE ɛ4 carriers compared with APOE ɛ4 non-carriers. Using structure MRI, Wang et al. (20) revealed that left hippocampal volumes of APOE ε4 carriers were smaller than those of APOE ε4 non-carriers. Numerous studies have found that brain function and structural damage in APOE ɛ4 carriers may affect their cognitive function compared with non-carriers, however, few studies have directly explored the differences in brain function and structure between APOE ɛ2 and APOE ɛ4 carriers. Therefore, directly exploring the differences in brain function and structure between APOE ɛ2 and APOE ɛ4 carriers may help us understand the reasons for the protective and impaired cognitive function associated with these two genotypes, the pathogenesis of AD, and discover novel cognitive-related imaging markers. However, APOE ɛ2 is a less common allele, the frequency is about 12% in Han Chinese or less (21), which makes it harder to study the differences between the two genotypes, this might be the reason for the fewer studies exploring the differences between carriers of APOE ɛ2 and ɛ4 alleles (7, 22, 23). In addition, a previous study has reported that APOE ε2/ε4 carriers still have a higher risk of AD (24). The cognitive impairment of APOE ε4 was much greater than the protective effect of APOE ε2 (25). The knowledge gained from previous studies makes us to speculate three assumptions about APOE ɛ2 and APOE ɛ4, meanwhile, rs-fMRI and structure MRI studies of these two genotypes have not been reported, especially in non-dementia elderly. we inferred that (i) cognition function of APOE ε2 carriers is higher than APOE ε4 carriers in non-dementia older, especially in the memory cognitive domain. (ii) Compared to subjects with APOE ε4, APOE ε2 carriers had increased rsFC in multiple brain networks, especially in DMN. (iii) Hippocampus volume of APOE ε4 carriers is reduced relative to APOE ε2 carriers obviously in structure MRI.

In this study, we examined the cognitive performance, resting-state networks (RSN) and grey matter (GM) between these two functionally opposite genotypes in elderly non-dementia carriers, and Independent component analysis (ICA) (26) and voxel-based morphometry (VBM) (27) were used. Finally, the correlation between differential brain regions after analysis and neuropsychological test scores were calculated.

## 2. Methods

### 2.1. Participants

A total of 530 right-handed Han Chinese participants from Qingdao City were enrolled in the study, as part of the sub-cohort in the Beijing Aging Brain Rejuvenation Initiative (BABRI) study (28). Only 63 subjects met the following criteria: (1) Mini-Mental Status Examination (MMSE) ≥24; (2) aged 50–80 years; (3) clinically diagnosed as non-demented patients by an experienced physician, and a clinical dementia rating (CDR) of 0; (4) no previous or current medical and neurological disorders to impact cognition; (5) no abnormality in the MRI of the brain structure and no any contraindication of MRI; and (6) blood samples were collected successfully for subsequent genotyping, APOE ε2 and ε4 carriers. The study was approved by the Ethics Committee of The Affiliated Hospital of Qingdao University, and signed informed consent was obtained from all subjects.

### 2.2. Neuropsychological testing

All subjects were evaluated using Mini-Mental state examination (MMSE) to assess general cognitive function and a battery of neuropsychological tests to assess five cognitive domains that included memory, attention, visuospatial ability, language, and executive function. Each cognitive domain used two different types of neuropsychological test scales, that tests were conducted by an experienced psychologist. Neuropsychological test scales were as follows. (1) Memory: the Auditory Verbal Learning Test (AVLT) and the Rey Osterrieth Complex Figure test (ROCF; recall). (2) Attention: the Trail Making Test (TMT-A) and the Symbol Digit Modalities Test (SDMT). (3) Visuospatial ability: ROCF (copy) and Clock Drawing Test (CDT). (4) Language: Category Verbal Fluency Test (CVFT) and the Boston Naming Test (BNT). (5) Executive function: the TMT-B and the Stroop Color and Word Test (SCWT-C). The raw test scores were used in this study.

Since MMSE was the most commonly used cognitive screening tool, the definitions of the other tests were mainly introduced here. AVLT: Firstly, playing 12 words, then subjects were asked to quickly recall words that were heard, and the test was repeated three times in succession, AVLT(N1-N3); Secondly, 5 min after completing AVLT(N1-N3), participants were asked to freely recall 12 words they had heard in the first step; Thirdly, 20 min after completing AVLT(N4), participants were asked to freely recall 12 words that they heard in the first step. The number of words correctly recalled is the score for each section; AVLT(N1-N3), AVLT(N4), and AVLT(N5) reflect Immediate recall (IR), short delay recall (DR), and long DR, respectively (29). ROCF: Show the ROCF standard figure to the participants, and tell them to copy it on the blank paper, the first 4 strokes with a red pen and the rest with a black pen, limited 5-10 min, ROCF(copy) (30). Record the completion time, and tell them to recall the figure after 20 min, same procedure as before, ROCF(recall) (30). TMT-A: In the TMT-A standard test paper, subjects were asked to connect from the number 1 in the order from small to large, and not lift the pen when connecting, the faster the better, and the limit of 5 min (31). TMT-B: In the TMT-B standard test paper, told the subjects to connect from the number 1 in accordance with the order from small to large, and pay attention to the outer border of the number, in accordance with the rule of “one square and one circle alternately.” Do not lift the pen when connecting and the faster the better. The duration of the TMT-A and TMT-B was the final score (31). SDMT: The subjects were informed that each number (1–9) corresponds to a different symbol, and the subjects were asked to fill in the symbol corresponding to the corresponding number in the SDMT standard test paper within 90 s, and the correct number was the total score (32). CDT: The subjects were asked to draw a watch face on the blank paper, and marked the numbers and hands, the time shown on the watch is 1:50, draw vertically, the first 6 strokes with a red pen, and the rest with a black pen (33). CVFT contains three parts, Animal fluency, Vegetable fluency, and Fruit fluency. Each test takes 1 min, and participants were asked to name as many animals, vegetables, or fruits per minute, as possible without repeating them, words repeated do not count toward the total score, and the sum of the three parts was the total score (34). BNT: A total of 30 BNT series of standard charts were shown to the subjects, and the subjects were asked what the pictures were, and the number of correct answers was the final score (35). SCWT includes three parts: A, B, and C. There were three cards with 50 items each and Card A is for reading Chinese characters printed in black (red, yellow, blue, and green), card B is for dots of four colors, and card C is for Chinese characters with inconsistent color and meaning. Each part of the test is evaluated by two indicators, namely time and the correct number. SCWT-A(time) and SCWT-A(correct), SCWT-B(time) and SCWT-B(correct), SCWT-C(time) and SCWT-C(correct), SCWT-C(time) and SCWT-C(correct) were extensively used to assess the ability to inhibit cognitive interference and executive function (36).

### 2.3. Genotyping

APOE DNA was extracted from blood samples of subjects that based on standard procedures and the PCR (Applied Biosystems, Foster City, CA) was used for subsequent genotype characterization. Two single nucleotide polymorphism genotypes of APOE were divided into rs429358 and rs7412 as previously described (37), and then APOE ε2 carriers and APOE ε4 carriers were differentiated based on rs429358 and rs7412. A total of 530 subjects were successfully genotyped. The proportion of each APOE genotype was as follows: ε2/ε2 (5/530, 0.94%), ε2/ε3 (64/530, 12.08%), ε2/ε4 (5/530, 0.94%), ε3/ε3 (367/530, 69.25%), ε3/ε4 (80/530, 15.09%), and ε4/ε4 (9/530, 1.70%). The frequency of ε2, ε3, and ε4 was 7.45, 82.83, and 9.72%, respectively. Only 63 subjects met the final inclusion and exclusion criteria. There were 32 APOE ε4 carriers (including 2 ε2/ε4, 26 ε3/ε4, and 4 ε4/ε4) and 31 APOE ε2 carriers (including 1 ε2/ε2 and 30 ε2/ε3).

### 2.4. MRI data acquisition

All participants were scanned with a General Electric (GE) sigma HDX 3.0 Tesla scanner at the Affiliated Hospital of Qingdao University, including BOLD and T1-weighted MRI scans. During scanning, noise-reducing headphones and foam padding were used to reduce head motion and noise impact. Moreover, all subjects were asked to stay awake and close their eyes during scanning. An echo-planar imaging (EPI) sequence was used to acquire rs-fMRI data: 33 axial slices, repetition time (TR) = 2,000 ms, echo time (TE) = 30 ms, slice thickness = 3.5 mm, flip angle = 90°, matrix = 64 × 64, 240 volumes and field of view (FOV) = 200 mm × 200 mm. High-resolution T1-weighted images were acquired using a magnetization-prepared rapid gradient-echo (MPRAGE) sequence: 176 sagittal slices, TR = 1,900 ms, TE = 3.44 ms, voxel size: 1 mm × 1 mm × 1 mm, acquisition matrix = 256 × 256, slice thickness = 1 mm, FOV = 256 × 256 mm (38).

### 2.5. Data preprocessing of rs-fMRI

RESTplus^1^ is a practical toolbox of rs-fMRI preprocessing based on Statistical parametric mapping version 12 (SPM12)^2^ and MATLAB2013b. RESTplus was used to preprocess all rs-fMRI data before statistical analysis. (1) Raw data was converted into a NIFTI format by dcm2nii.^3^ (2) The first 10 points were removed from all time points to dispel noise impact and acquire more stable data. (3) Slice timing correction was implemented, which means the remaining 230 volumes were corrected for the acquisition time difference between slices. (4) To correct possible head motion during the rs-fMRI data acquisition, the between-frame realignment was performed the head motion parameter of frame displacement (FD) to be included in the statistical model was computed to further account for the movement effect. Translational or rotational motion parameters were less than 2 mm or 2°, respectively. (5) Spatial normalization of T1-weighted images to a Montreal Neurological Institute (MNI) template space was performed using the DARTEL algorithm and resampled to 3 mm x 3 mm voxels. (6) The final preprocessing step before ICA is Gaussian smoothing, which was applied to smooth the data based on the full-width at half maximum (FWHM) [6 6 6] for normal distribution.

### 2.6. Determination of resting state networks

**Figure 1:**
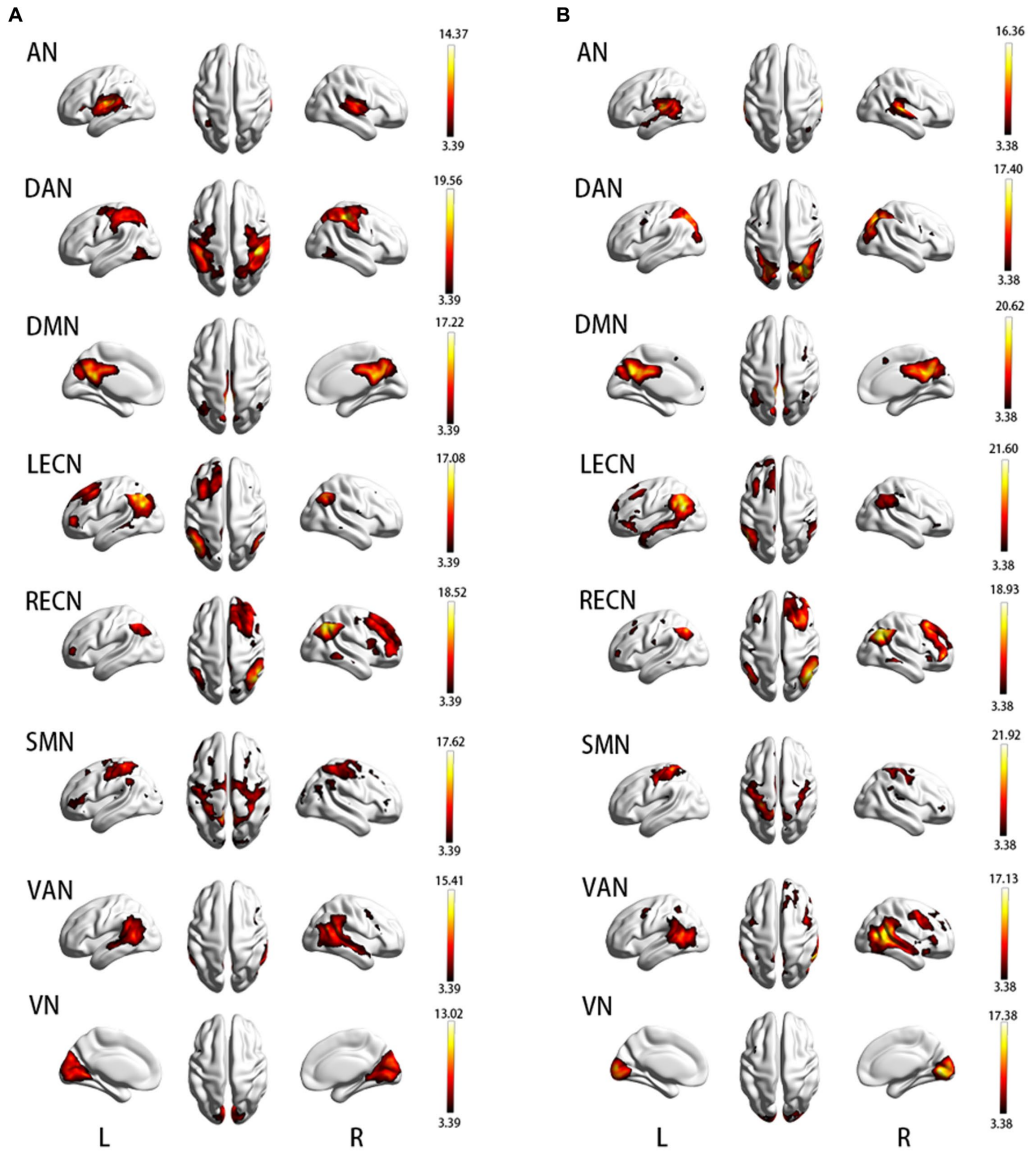
**(A)** Resting-state brain network map in APOE ε2 carriers; **(B)** Resting-state brain network map in APOE ε4 carriers. AN, auditory network; DAN, dorsal attention network; DMN, default mode network; LECN, left executive control network; RECN, right executive control network; SMN, somatomotor network; VAN, ventral attention network; VN, visual network.

In short, ICA like a machine of classification can sort out some synchronously activated brain regions according to the similarity of BOLD signals. These brain regions cooperate in the resting state and thus are called RSNs (15, 17, 39). ICA is a method used to obtain components with high temporal similarity and elevated spatial similarity. The Group ICA/IVA of fMRI toolbox (GIFT V3.0b, https://trendscenter.org/software/) based on MATLAB2013b was used to obtain independent components (ICs) of APOE ε2 and ε4 carriers, respectively. Processing steps were as follows (1) “Minimum description length” criterion was used to estimate the number of ICs of the two groups, respectively. For every subject, the estimated components were calculated, and the mean of all the estimated components was taken. Thirty-five ICs were identified in APOE ε2 carriers and 39 ICs were identified in APOE ε4 carriers. (2) The “Infomax algorithm” was used to calculate the ICs of the two groups, respectively. “ICASSO” was used to carry out group-ICA. ICA was run 100 times and the best estimate for each component is used. (3) All steps were run, including Parameter Initialization, Data Reduction, Calculate ICA, Back Reconstruction, Calibrate Components, and Group Stats. Finally, the 35 ICs and 39 ICs of the two groups were obtained. ICs consist of the spatial map that represents brain activity intensity of voxel and the time course that represents the waveform of brain activity, which was extracted from every subject. Lastly, the intensity values of the spatial map were converted to Z-values (40) that is deemed to be the mightily effective way to measure the rsFC of intranetwork (41) as well as spatial maps was used to screen RSNs. Two complementary approaches were used in to identify the RSNs (42): (1) calculation of the correlation coefficient based on GIFT between ICs and the RSN template (FIND Lab at Stanford University), the maximum spatial correlation coefficient is considered strong evidence for selected ICs and (2) visual screening and inspection of which brain region similarity between the obtained ICs and RSN templates. Using the two approaches described above, we obtained eight RSNs that included Auditory Network (AN), Dorsal Attention Network (DAN), Default Mode Network (DMN), Left Executive Control Network (LECN), Right Executive Control Network (RECN), Somatomotor Network (SMN), Ventral Attention Network (VAN), and Visual Network (VN). The one-sample t-test and cluster-wise family-wise error (FWE) correction (*p* < 0.05) were carried out for each RSN of the two groups to determine the true active brain regions. The spatial map of RSNs is shown in Figure 1. An “Image Calculator” in SPM12 was used to calculate the intersection mask that limits the scope of intra-network analysis between the two groups.

### 2.7. Data preprocessing of structure MRI

VBM8 is a widely accepted toolbox based on SPM8 (See text footnote 2) in the field of neuroimaging research to characterize the regional gray matter volume (GMV). VBM8 was used to compute the GMV of all subjects (27). As previously described, the raw image was converted into a NIFTI format by dcm2nii. (See text footnote 3) The CheckReg tool in SPM8 was used to check the image quality to ensure that there was no obvious abnormality in the T1-weighted image. T1-weighted images were normalized to the MNI space using the DARTEL algorithm in VBM8, then the bias field correction was performed to prepare for segmentation. The image quality was checked again. Next, the image was segmented into GMV, white matter volume (WMV), and cerebrospinal fluid (CSF), which together make up the total intracranial volume (TIV). Finally, the image was modulated and smoothed with FWHM [8 8 8] before further statistical analysis.

### 2.8. RSFC analyses and GMV analyses between two groups

In the rsFC analyses, two-sample t-test based on SPM12 was used to compare the difference of rsFC in each RSNs map between the two groups, that is intra-network analyses. AN of APOE ε2 carriers compared with AN of APOE ε4 carriers, and so on. Previously calculated intersection mask was used to limit the statistic range and age, gender, and education level as a covariate. The final result uses cluster-wise FWE correction (voxel-wise *p* < 0.001 and cluster-wise *p* < 0.05) to remove false positive results. Regions of the brain where ε2 carriers have higher rsFC in all brain networks than ε4 carriers are defined as ROI-A and where ε2 carriers had lower rsFC in all brain networks than ε4 carriers were defined as ROI-B.

In GMV analyses, two-sample t-test based on SPM8 was used to compare the difference of the whole brain in GMV between the two groups, age, gender, education level and TIV as covariates, GM mask was used to limit the statistic range in the whole brain. Cluster-wise FWE correction (voxel-wise *p* < 0.001 and cluster-wise *p* < 0.05) is also used for the final result. After GMV analyses of the whole brain, differential brain regions were considered as ROIs, respectively.

### 2.9. Statistical analysis

The two-sample t-test was utilized to analyze differences in age, education, and neuropsychology test between the two groups, and the Chi-square test was used to analyze the gender difference. Data were analyzed using IBM SPSS Statistics 23.0 software (IBM Corp., Armonk, NY), and *p* < 0.05 was considered statistically significant. The values of ROIs after intranet-work rsFC analyses and GMV analyses of the whole brain were extracted by RESTplus utilities “extracted regions of interest (ROI) signals,” and then SPSS 23.0 was used to calculate the Partial correlation coefficients between all neuropsychological test scores and ROIs based on the whole sample and controlling age, gender, and education level. In multiple Partial correlation analysis, false discovery rate (FDR) correction based on the function of Matlab2013b was used to control false positives, FDR value<0.05 was considered as significance.

## 3. Results

### 3.1. Demographic and neuropsychological test

No significant differences in age, gender, and years of education were found between the two groups (*p* > 0.05). Demographic and neuropsychological test scores data are summarized in Table 1. Although all subjects were from a non-dementia population, APOE ε2 carriers still had higher MMSE scores in general cognitive situations, compared with APOE ε4 carriers (*p* < 0.001). Moreover, both AVLT (N1-N3) and ROCF (recall) showed significant differences in the two memory cognitive domain tests (*p* < 0.05). The first assumption proposed previously is that the cognition function of APOE ε2 carriers is higher than APOE ε4 carriers in non-dementia older, especially in the memory cognitive domain, which was verified. The SDMT test of the attention cognitive domain and the SCWT-C (time) test of the executive function cognitive domain also showed significant differences between the two groups (*p* < 0.05). No difference in the language cognitive domain and the visuospatial cognitive domain was found between ε2 and ε4 carriers (*p* > 0.05).

**Table 1:** Demographic and neuropsychological data of the all subjects.

	ε2 carriers (*n* = 31)	ε4 carriers (*n* = 32)	statistics	*p*-value
Male/Female	12/19	11/21	χ2 = 0.128	0.721^b^
Age (years)	62.19 ± 8.36 (50–76)	65.25 ± 7.28 (51–78)	−1.549	0.127^a^
Education (years)	11.50 ± 3.60 (2–20)	11.50 ± 3.94 (4–21)	0.000	1.000^a^
General mental status				
MMSE	28.07 ± 1.84 (24–30)	26.13 ± 2.00 (24–30)	4.253	<0.001^a^
Memory				
AVLT(N1-N3)	17.52 ± 5.10 (2–27)	14.34 ± 6.20 (3–28)	2.125	0.031^a^
AVLT(N4)	5.16 ± 2.46 (1–11)	3.88 ± 3.58 (0–12)	1.656	0.103^a^
AVLT(N5)	4.16 ± 2.45 (0–9)	3.47 ± 3.52 (0–12)	0.904	0.370^a^
ROCF(recall)	14.77 ± 8.53 (1–34)	9.63 ± 8.59 (0–34)	2.386	0.020^a^
Attention				
SDMT	37.29 ± 14.66 (13–66)	28.47 ± 11.55 (2–53)	2.658	0.010^a^
TMT-A	57.03 ± 27.28 (26–138)	82.65 ± 69.95 (29–438)	−1.904	0.062^a^
Visuospatial				
CDT	23.10 ± 5.17 (14–30)	21.13 ± 7.85 (2–29)	1.174	0.245^a^
ROCF (copy)	33.65 ± 4.42 (18–38)	30.47 ± 9.12 (2–36)	1.749	0.085^a^
Language				
CVFT	43.55 ± 8.06 (17–57)	38.59 ± 11.53 (22–74)	1.971	0.053^a^
BNT	23.55 ± 4.13 (14–30)	21.97 ± 4.58 (12–28)	1.437	0.156^a^
Executive function				
TMT-B	151.42 ± 59.29 (67–316)	177.16 ± 53.94 (92–326)	−1.803	0.076^a^
SCWT-C (time)	79.94 ± 25.25 (48–150)	99.63 ± 38.51 (29–223)	−2.392	0.020^a^
SCWT-C (correct)	46.97 ± 3.43 (35–50)	45.03 ± 5.78 (21–50)	1.610	0.113^a^

The raw scores of all neuropsychological were shown as the mean ± standard deviation. The data range was also provided in parentheses. MMSE, mini-mental state examination; AVLT, auditory verbal learning Test; ROCF, Rey-Osterrieth complex figure; SDMT, symbol digit modalities Test; TMT, trail making test; SCWT, stroop color, and word test; CVFT, category verbal fluency test. BNT, boston naming test. CDT, clock drawing test.

a The *p*-values were obtained by two-sample t-test.

b The *p*-value was obtained by the chi-square test.

### 3.2. Difference in RSNs between the two groups

**Figure 2:**
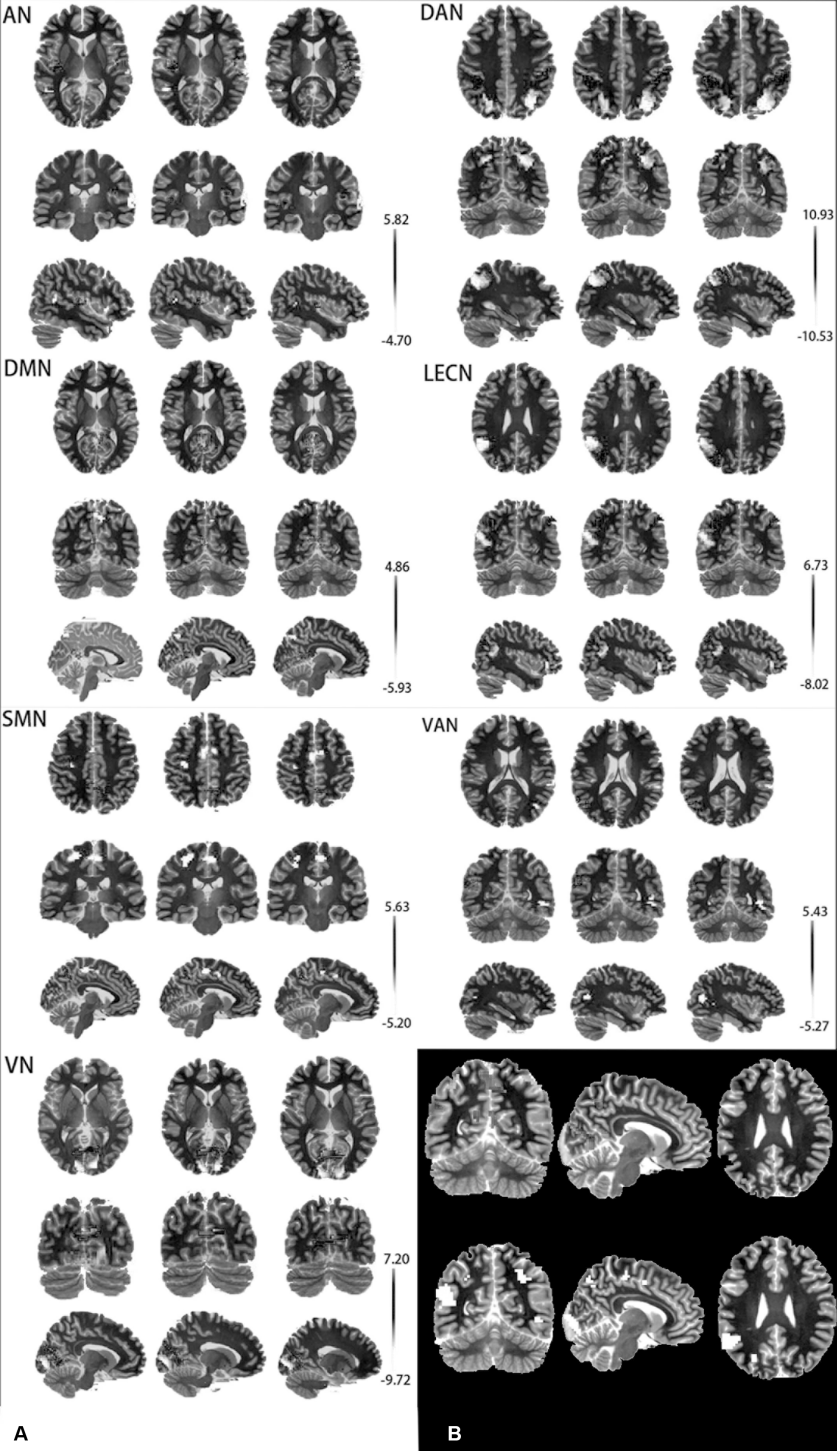
**(A)** Differential brain regions of rsFC between APOE ε2 carriers and APOE ε4 carriers in 7 resting-state brain networks. **(B)** Red: ROI-A; Blue: ROI-B; AN, auditory network; DAN, dorsal attention network; DMN, default mode network; LECN, left executive control network; SMN, somatomotor network; VAN, ventral attention network; VN, visual network.

A two-sample t-test and cluster-wise FWE correction showed a significant difference in brain regions of AN, DAN, DMN, LECN, SMN, VAN, and VN (*p* < 0.05) and no difference in RECN. The difference in the brain regions of the two groups is shown in Figure 2, and detailed information on differential brain regions after the correction is summarized in Table 2. In the AN, compared with ε4 carriers, ε2 carriers exhibited increased rsFC in the left Heschl’s gyrus (HES.L), right Rolandic operculum (ROL.R), and decreased rsFC in the right superior temporal gyrus (STG.R), left middle temporal gyrus (MTG.L). In the DAN, ε2 carriers presented increased rsFC in the bilateral inferior parietal, but supramarginal and angular gyrus (IPL.L, IPL.R), and decreased rsFC in the left superior occipital gyrus (SOG.L), right middle occipital gyrus (MOG.R) relative to ε4 carriers. In DMN, compared with ε4 carriers, ε2 carriers exhibited increased rsFC in the left calcarine fissure and surrounding cortex (CAL.L), and decreased rsFC in the left cuneus (CUN.L), right precuneus (PCUN.R). In the LECN, ε2 carriers presented increased rsFC in the IPL.L, right angular gyrus (ANG.R), left middle frontal gyrus (MFG.L) and decreased rsFC in the MTG.L, left inferior frontal gyrus, orbital part (ORBinf.L) relative to ε4 carriers. In the SMN, compared with ε4 carriers, ε2 carriers exhibited increased rsFC in the left precuneus (PCUN.L), and decreased in the left precentral (PreCG.L), left supplementary motor area (SMA.L). In the VAN, ε2 carriers presented increased rsFC in the left supramarginal gyrus (SMG.L) and decreased rsFC in the right inferior temporal gyrus (ITG.R), right middle temporal gyrus (MTG.R), and right superior temporal gyrus (STG.R) relative to ε4 carriers. In the VN, compared with ε4 carriers, ε2 carriers exhibited increased rsFC in the left lingual gyrus (LING.L) and decreased rsFC in the right calcarine fissure, and surrounding cortex (CAL.R).

**Table 2:** Information of the brain regions after rsFC analyses.

ROI	RSN	Brain regions	Cluster size	Coordinates (X Y Z)	Peak T-value
A	AN	HES.L	34	−48 −12 9	5.8163		ROL.R	24	48 −9 12	5.3038	DAN	IPL.L	131	−39 −48 57	7.595		IPL.R	166	48 −33 48	10.9269	DMN	CAL.L	82	−6 −63 15	4.8612	LECN	IPL.L	112	−36 −78 42	6.7337		ANG.R	22	48 −63 45	5.3185		MFG.L	40	−27 24 54	6.2092	SMN	PCUN,L	61	−3 −60 57	5.6261	VAN	SMG.L	49	−57 −51 27	5.4305	VN	LING.L	292	−9 −63 6	7.2004
B	AN	STG.R	64	63 −24 12	−5.2101		MTG.L	15	−48 −51 9	−4.6516	DAN	SOG.L	131	−21 −75 33	−7.1761		MOG.R	258	33 −75 42	−10.5267	DMN	CUN.L	17	−6 −78 36	−4.3115		PCUN.R	34	6 −69 48	−5.926	LECN	MTG.L	36	−60 −24 −6	−5.4793		ORBinf.L	19	−42 33 –3	−5.8723		MTG.L	215	−57 −48 24	−8.0241	SMN	PreCG.L	63	−24 −27 60	−5.0144		SMA.L	133	−6 −6 54	−5.1957	VAN	ITG.R	42	48 −57 −6	−4.7342		MTG.R	55	36 −69 15	−5.2715		STG.R	33	57 −36 15	−5.0471	VN	CAL.R	318	15 −93 0	−9.7158

ROI-A, Brain regions with increased rsFC in each brain networks in ε2 carriers; ROI-B, Brain regions with decreased rsFC in each brain networks in ε2 carriers. AN, Auditory Network, DAN, Dorsal Attention Network, DMN, Default Mode Network, LECN, Left Executive Control Network, SMN, Somatomotor Network, VAN, Ventral Attention Network, VN, Visual Network. HES.L, left Heschl’s gyrus; ROL.R, right Rolandic operculum; STG.R, right superior temporal gyrus; MTG.L, left middle temporal gyrus; IPL, inferior parietal, but supramarginal and angular gyrus; SOG.L, left superior occipital gyrus; MOG.R, right middle occipital gyrus; CAL.L, left calcarine fissure and surrounding cortex; CUN.L, left cuneus; PCUN.R, right precuneus; ANG.R, right angular gyrus; MFG.L, left middle frontal gyrus; ORBinf.L, left inferior frontal gyrus, orbital part; PCUN.L, left precuneus; PreCG.L, left precentral; SMA.L, left supplementary motor area; SMG.L, left supramarginal gyrus; ITG.R, right inferior temporal gyrus; MTG.R, right middle temporal gyrus; STG.R, right superior temporal gyrus; LING.L, left lingual gyrus; CAL.R, right calcarine fissure, and surrounding cortex.

### 3.3. Correlation between neuropsychological tests and ROIs

**Figure 3:**
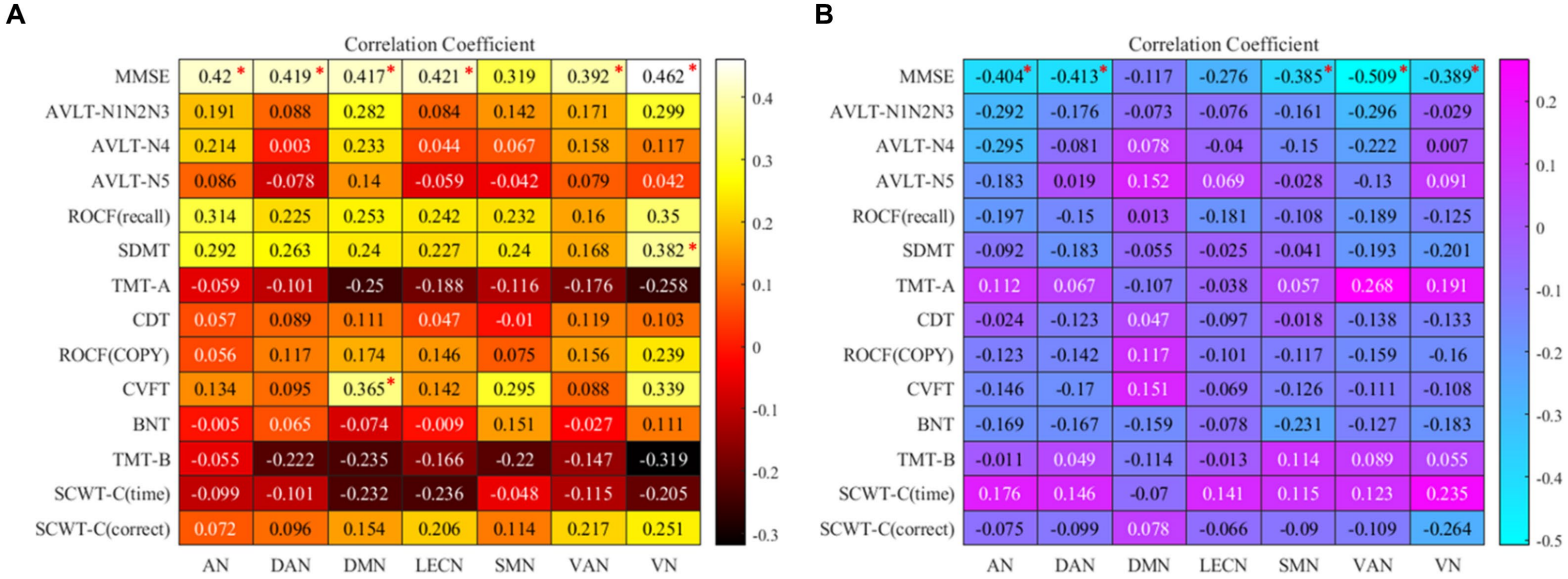
**(A)** the map of Correlation coefficients between ROI-A and neuropsychological tests. **(B)** The map of Correlation coefficients between ROI-B and neuropsychological tests. “*”: the correlation coefficient has been corrected by FDR; AN, auditory network; DAN, dorsal attention network; DMN, default mode network; LECN, left executive control network; SMN, somatomotor network; VAN, ventral attention network; VN, visual network. MMSE, mini-mental state examination; AVLT, auditory verbal learning test; ROCF, Rey-Osterrieth complex figure; SDMT, symbol digit modalities test; TMT, trail making test; SCWT, Stroop color and word test; CVFT, category verbal fluency test; BNT, boston naming test; CDT, clock drawing test.

In the partial correlation analysis between ROI-A and neuropsychological after controlling for age, gender, education, and FDR correction, the brain region with rsFC increased in AN, DAN, DMN, LECN, VAN, and VN were positively correlated with MMSE. The brain region with rsFC increased in VN was positively correlated with SDMT, and the brain region with rsFC increased DMN was positively correlated with CVFT. In the partial correlation analysis between ROI-B and neuropsychological after controlling for age, gender, education, and FDR correction, he brain region with rsFC decreased in AN, DAN, SMN, VAN, and VN were negatively correlated with MMSE. Detailed information was summarized in Figure 3.

### 3.4. Difference in the GMV and the correlation between neuropsychological tests and ROIs

**Figure 4:**
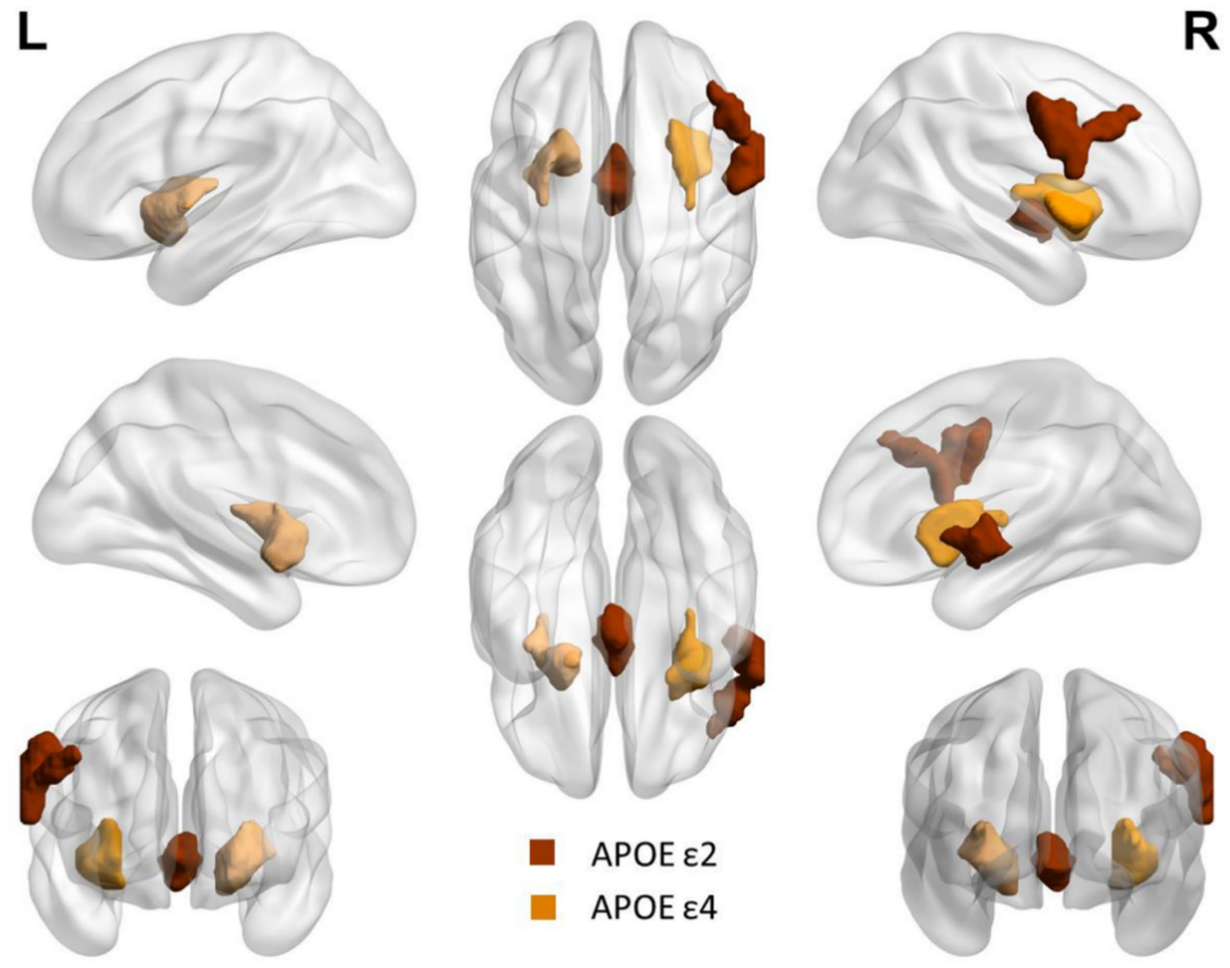
Differential brain regions between APOE ε2 carriers and APOE ε4 carriers after GMV analysis. Red: thalamus and right postcentral gyrus. Yellow: bilateral putamen.

**Figure 5:**
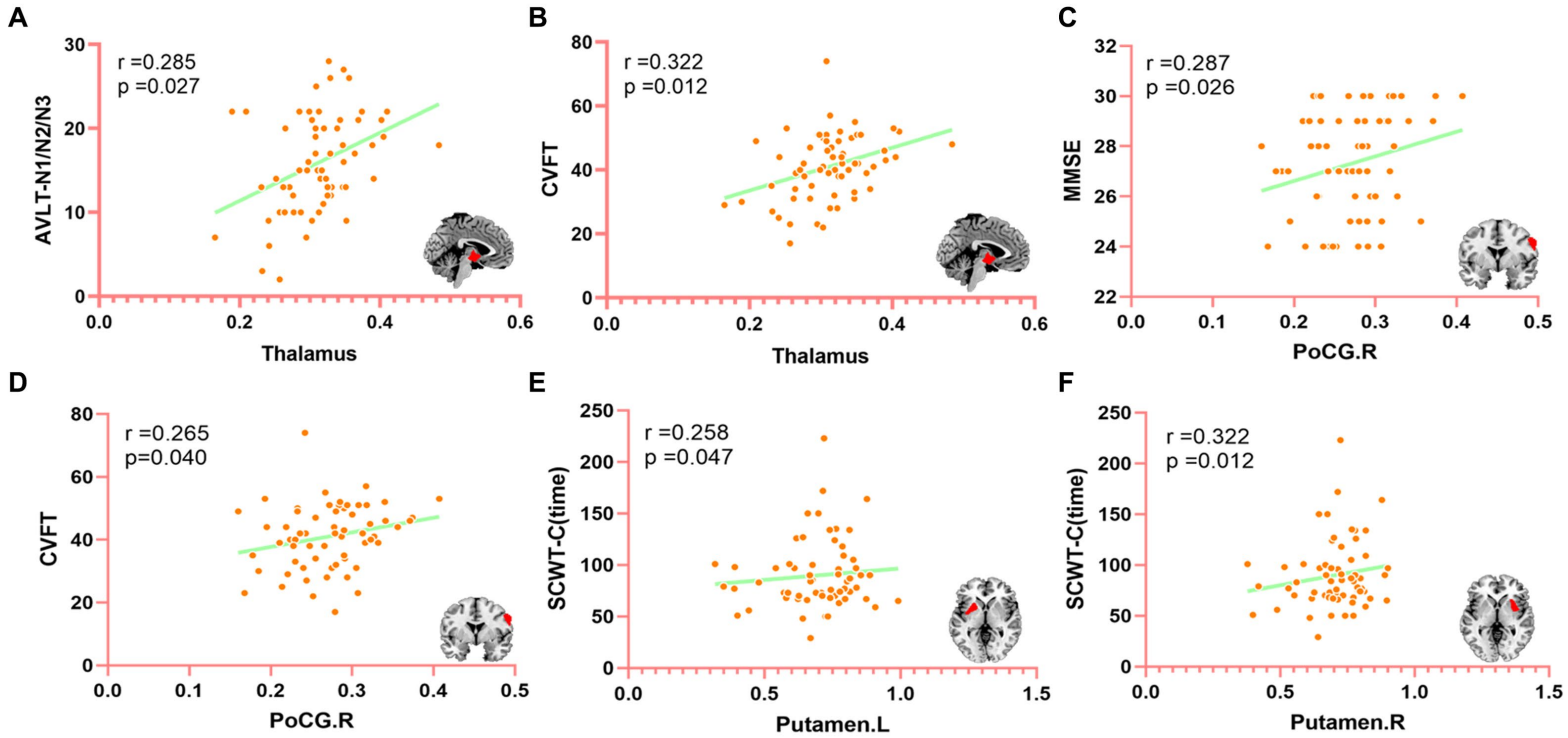
The correlation between differential brain regions after GMV analysis and neuropsychological (controlling age, gender, and education of partial correlation analysis). MMSE, mini-mental state examination; CVFT, category verbal fluency test; AVLT, auditory verbal learning test; SCWT, Stroop color and word test. PoCG.R, right posterior central gyrus. **(A)** The correlation between the thalamus and AVLT (N1-N3), **(B)** The correlation between the thalamus and CVFT, **(C)** The correlation between PoCG.R and MMSE, **(D)** The correlation between PoCG.R and CVFT, **(E)** The correlation between Putamen. L and SCWT-C (time), and **(F)** The correlation between Putamen.R and SCWT-C (time).

Compared with APOE ε4 carriers, APOE ε2 carriers had larger volumes of the thalamus and the right postcentral gyrus (PoCG.R). The volume of bilateral putamen of APOE ε4 carriers was larger than that of APOE ε2 carriers. The detailed coordinates and cluster size of the corrected differential brain regions are shown in Table 3, and the location of the brain regions is shown in Figure 4. After GMV analyses of the whole brain between the two groups, these differential brain regions between two groups were defined as ROIs. Next, we extracted values of ROIs, respectively, and then calculated the Partial correlation with neuropsychological test scores, after controlling the age, gender, and education. Results showed that the volume of the thalamus was positively correlation with AVLT (N1-N3; r = 0.285, *p* = 0.027), and CVFT (r = 0.322, *p* = 0.012; Figures 5A,B). The volume of the PoCG.R was positively correlation with MMSE (r = 0.287,*p* = 0.026) and CVFT(r = 0.265,*p* = 0.040; Figures 5C,D). The volume of the left putamen was positively correlation with SCWT-C(time; r = 0.258,*p* = 0.047; Figure 5E). The volume of the right putamen was positively correlation with SCWT-C (time; r = 0.322,p = 0.012; Figure 5F).

**Table 3:** Information of the brain regions after GMV analyses.

Brain regions	Cluster size	Coordinates (X Y Z)	Peak T-value
Thalamus	1,211	−1.5 −7.5 −7.5	5.2867
PoCG.R	1,734	58.5–1.5 34.5	4.6252
Putamen.R	1,719	−30 −4.5 7.5	−4.4479
Putamen.L	1,454	31.5 1.5 6	−4.7141

PoCG.R: right postcentral gyrus. L:left; R:right.

## 4. Discussion

### 4.1. Differences in brain areas based on rs-fMRI

The ICA was used to assess the differences in RSNs between carriers of the cognitively protective APOE ε2 allele and carriers of the deleterious APOE ε4 allele. We found that in the seven differential RSNs, APOE ε2 carriers had 10 brain regions with increased rsFC and 14 brain regions with decreased rsFC (removing the overlapping brain regions). Compared with APOE ε4 carriers, APOE ε2 carriers showed not many brain regions with increased rsFC in multiple brain networks, especially in DMN, SMN, and VAN. This result is not consistent with our second assumption that APOE ε2 carriers with better cognition would have more brain regions with increased rsFC, particularly in DMN. An ICA study of patients with mild cognitive impairment (MCI) and normal controls found fluctuations in multiple brain networks between patients with MCI and normal controls, with normal controls showing no more areas of increased rsFC compared with MCI patients (42). Some researchers argued that people with better cognitive function are able to economize on brain use and show better cognitive performance (43). This might be the reason why APOE ε2 carriers had a superior cognitive performance relative to APOE ε4 carriers, though, the brain regions of increased rsFC are less than APOE ε4 carriers. However, different brain regions have particular functions, for instance, Heschl’s gyrus. A study on rs-fMRI of musicians found that the rsFC of Heschl’s gyrus of musicians was significantly higher than that of normal people, which may explain why musicians are better at processing some sounds than normal (44). Therefore, in our study, all differences in brain regions were evaluated in combination with cognitive performance between the two groups.

We individually calculated the correlation between ROI-A, ROI-B, and neuropsychological tests, Figure 3. In the Partial correlation analysis of ROI-A and neuropsychological tests, after controlling the age, gender, and education, we found in AN, DAN, DMN, LECN, VAN, and VN, the brain regions with increased rsFC were positively correlated with MMSE. From the perspective of the correlation coefficient, in addition to the weak correlation between VAN and MMSE, AN, DAN, DMN, LECN, and VN and MMSE all represent moderate correlation, which suggests that these brain regions with increased rsFC may lead to better general cognitive function and it may also be one of the reasons that the MMSE score of ε2 carriers is significantly higher than that of ε4 carriers (*p* < 0.001). In DMN, the brain region with increased rsFC was positively correlated with CVFT that was used to assess the language, and we could see that the average CVFT score of ε2 carriers was higher than that of ε4 carriers in Table 1, though there was no significant (*p* = 0.053). These results reflected that while the brain region with increased rsFC leads to better language, it might be not enough. In VN, the brain region with increased rsFC was positively correlated with SDMT which was used to assess attention. In SDMT, the subjects need constantly scanned the test paper to obtain symbols that match the numbers, and the brain regions associated with vision are highly active during this process, and concentration. Therefore, the brain region with increased rsFC in VN might be a reason why the SDMT score of ε2 carriers was higher than ε4 carriers (*p* = 0.01). In the Partial correlation analysis of ROI-B and neuropsychological tests, after controlling the age, gender, and education. In AN, DAN, SMN, VAN, and VN, these brain regions with decreased rsFC were negatively correlated with MMSE. However, the increased rsFC and decreased rsFC were relative between the two groups, thus, these brain regions with decreased rsFC in ε2 carriers were also the brain regions with rsFC increased in ε4 carriers. These brain regions were negatively correlated with MMSE which also explains why the MMSE score of ε4 carriers was lower than that of ε2 carriers. Combined with these results, it is not difficult to find that the brain regions with increased rsFC in ε2 carriers bring better cognitive performance to ε2 carriers, and the brain regions with increased rsFC in ε4 carriers make the cognitive performance of ε4 carriers worse. This may be a mechanism for the brain to compensate for cognitive deficits (42). Cognitive impairment does not necessarily result in fewer activated brain areas, but these activated brain areas are not associated with better cognition and may be compensated for poorer cognitive performance (43).

### 4.2. Difference in brain areas based on structure MRI

GMV analysis of the whole brain showed that the thalamic and PoCG.R volume of APOE ε2 carriers was higher than that of APOE ε4 carriers; however, the bilateral putamen volume of APOE ε4 carriers was higher than that of APOE ε2 carriers. Although there were significant differences in the performance of the two groups in the memory cognitive domain, no difference in bilateral hippocampus volume was found between the two groups. The third assumption proposed previously is that the hippocampus volume of APOE ε4 carriers is reduced relative to APOE ε2 carriers, which was not verified. The thalamus plays a key role both non-specific projection systems and specific projection systems (45). The primary sensory cortex is located in the postcentral gyrus, the main function is to receive somatic sensations from the opposite half of the body. A previous study has also shown that the postcentral gyrus plays an essential role in emotion regulation (46). The thalamus and postcentral gyrus are the two major stations in the superficial sensory pathway, superficial sensory neurons are replaced in the thalamus and then finally project to the postcentral gyrus to produce a specific sensory (45, 46). Asami et al. (47) found that compared to healthy control, female patients with panic disorder had substantial reductions in the GMV in the thalamus bilaterally in a voxel-wise volume comparison analysis, they argued that the reduction in the thalamic volume is associated with emotion regulation and cognitive function. APOE ε2 carriers had higher thalamic and PoCG.R volumes compared with APOE ε4 carriers, suggesting that APOE ε2 carriers might have more advantages in the processing of body surface sensation and emotion regulation compared with APOE ε4 carriers. However, a longitudinal VBM study by Squarzoni et al. (48) reported a reduced thalamic volume and hippocampal region in elderly healthy adults with the APOE ɛ4 non-carriers, and they argued that the reduction in the thalamic volume was not associated with APOE ɛ4. Our results are not consistent with Squarzoni’s finding, which might be due to the different research methods and different participants.

Using fluorodeoxyglucose (FDG)-positron emission tomography (PET), Klunk et al. (49) found extremely early amyloid deposition in the striatum, including putamen and caudate, in a high-risk Alzheimer’s disease population, and this change was not associated with clinical symptoms. Using a similar method to that of Klunk and colleagues, Pardo and Lee (50) also found that peak amyloid deposition localized principally to the putamen compared with other brain areas in non-dementia APOE ε4 homozygotes. Kapoulea and Murphy (51) used the structural MRI data and VBM-method and discovered that APOE ε4 carriers among non-dementia older population possessed a larger volume of bilateral putamen compared with APOE ε4 non-carriers but this change was not associated with cognitive performance. Kumar et al. (52) discovered that newly-diagnosed patients with obstructive sleep apnea had a larger bilateral putamen volume than healthy control, which they believed was due to chronic hypoxia of obstructive sleep apnea patients. Compared with APOE ε2 carriers, APOE ε4 carriers had a larger volume of bilateral putamen. These results might have an explanation based on our findings and those from previous studies. It might be that additional amyloid was deposited in bilateral putamen in APOE ε4 carriers (49–51), because APOE ε4 allele possesses a high risk for amyloid deposition (1, 53). Conversely, the APOE ε2 allele had a low risk for amyloid deposition (6, 7, 23). Therefore, the different sensitivity of APOE ε2 and ε4 carriers to the amyloid deposition could be an explanation for these results.

Finally, we calculated the Partial correlation between neuropsychological test scores and the value of ROIs after controlling the age, gender, and education to evaluate the impact of changed brain regions on cognitive function. We found that the thalamic volume positively correlated with AVLT (N1-N3) and CVFT, PoCG.R positively correlated with MMSE and CVFT, left putamen and right putamen positively correlated with SCWT-C(time). These results might indicate that the thalamus plays a role in IR and language. Fislage et al. (54) computed the correlation between the thalamic volume and positive screening scales of postoperative delirium and found that a larger thalamic volume was associated with reduced odds of postoperative delirium. Zidan et al. (55) inferred that thalamic volume loss could be an early sign of poorer cognitive performance in amnestic MCI. Our finding is consistent with data from previous studies, in which the thalamus plays a key role in cognitive performance (45, 48). PoCG.R plays a role in general mental status and language, and may also be one of the reasons why the MMSE score of ε2 carriers is significantly higher than that of ε4 carriers. Although bilateral putamen was positively correlated with SCWT-C(time), this was not a good thing in terms of the definition of SCWT-C(time). The SCWT was used to assess the ability to inhibit cognitive interference, the longer SCWT-C(time) means the worse ability to inhibit cognitive interference (36). This suggests that larger putamen volume corresponds to longer SCWT-C (time), indicating that the increase in putamen volume is not independent of cognition and may lead to worse executive function, though this is not consistent with Klunk et al. (49) and Kapoulea and Murphy (51) findings. The bilateral putamen volume of ε2 carriers is smaller than that of ε4 carriers, which may be the reason for the significant difference in SCWT-C(time) between the two groups. Taken together, the larger thalamus of ε2 carriers and the volume of PoCG.R gave ε2 carriers more advantages in general cognition, IR, and language, however, the larger putamen volume of ε4 carriers actually made their cognitive performance worse. It might be that for ε4 carriers with poor cognition, the function and volume of some brain regions are not all lower or smaller than that of ε2 carriers with higher cognition, but these brain regions are not associated with better cognitive performance. Whether these results are caused by the different regulation of the same pathology by the two genotypes of ε2 and ε4 needs to be further verified in the future, but these results may explain to some extent why ε2 carriers have better cognitive function than ε4 carriers.

## 5. Conclusion

In summary, the present study explored the differences in cognitive performance, brain structure, and function between the cognitively protective APOE ε2 allele and cognitively impaired APOE ε4 allele in non-dementia elderly carriers. It was found that cognitive differences between APOE ε2 and ε4 carriers are closely related to functional and GM differences, which are likely to cause cognitive differences between APOE ε2 and ε4 carriers. Therefore, a better understanding of the protective effects of the APOE ε2 allele and the deleterious effects of the APOE ε4 allele on human cognitive function is equally important. While these findings may be helpful in future clinical work, further research is needed to determine whether the thalamus, putamen, and PoCG.R could be potential imaging markers for the diagnosis of cognitive decline.

## Data availability statement

The raw data supporting the conclusions of this article will be made available by the authors, without undue reservation.

## Ethics statement

The studies involving humans were approved by The Ethics Committee of The Affiliated Hospital of Qingdao University. The studies were conducted in accordance with the local legislation and institutional requirements. The participants provided their written informed consent to participate in this study.

## Author contributions

ZW and JP: conceptualization, methodology, formal analysis, writing – original draft, and visualization. RZ, JQ, XS, BH, XM, and QW: data curation, investigation, methodology, project administration, software, and validation. JS: conceptualization, methodology, resources, supervision, project administration, and funding acquisition. All authors contributed to the article and approved the submitted version.

## Funding

This study is supported by the National Key R&D Program of China (2018YFC1315200).

## Conflict of interest

The authors declare that the research was conducted in the absence of any commercial or financial relationships that could be construed as a potential conflict of interest.

## Publisher’s note

All claims expressed in this article are solely those of the authors and do not necessarily represent those of their affiliated organizations, or those of the publisher, the editors and the reviewers. Any product that may be evaluated in this article, or claim that may be made by its manufacturer, is not guaranteed or endorsed by the publisher.

## Footnotes

^1^ http://restfmri.net/forum/RESTplus

^2^ http://www.fil.ion.ucl.ac.uk/spm

^3^ www.nitrc.org/projects/dcm2nii/

## Supplementary material

SUPPLEMENTARY TABLE 1 Details of APOE e2 carriers

SUPPLEMENTARY TABLE 2 Details of APOE e4 carriers

SUPPLEMENTARY TABLE 3 Brain network differences between the two groups Details of brain regions

SUPPLEMENTARY TABLE 4 ROI-A extract values

SUPPLEMENTARY TABLE 5 ROI-B extract values

SUPPLEMENTARY TABLE 6 Differential brain area extraction after GMV analysis

SUPPLEMENTARY TABLE 7 Details of brain regions with gray matter differences between the two groups
