# Corrigendum: Differences in resting-state brain networks and gray matter between APOE ϵ2 and APOE ϵ4 carriers in non-dementia elderly

**DOI:** 10.3389/fpsyt.2024.1365662

**Published:** 2024-01-22

**Authors:** Zhiyuan Wang, Jing Pang, Ruizhi Zhou, Jianjiao Qi, Xianglong Shi, Bin Han, Xu Man, Qingqing Wang, Jinping Sun

**Affiliations:** ^1^ Institute of Integrative Medicine, The Affiliated Hospital of Qingdao University, Qingdao, China; ^2^ Department of Radiology, The Affiliated Hospital of Qingdao University, Qingdao, China; ^3^ Department of Emergency Medicine, The Affiliated Hospital of Qingdao University, Qingdao, China; ^4^ Department of Neurology, The Affiliated Hospital of Qingdao University, Qingdao, China

**Keywords:** resting-state functional magnetic resonance imaging, APOE ϵ2, APOE ϵ4, independent component analysis, voxel-based morphometry, non-dementia elderly

In the published article, there was an error in the **Abstract**. The sentence previously stated:

“Finally, differences in brain function and structure may be might be the reason that APOE ϵ2 carriers are better than APOE ϵ4 carriers in cognitive performance”.

The corrected sentence appears below:

“Finally, differences in brain function and structure may be the reason that APOE ϵ2 carriers are better than APOE ϵ4 carriers in cognitive performance”.

In the original article, there was an error in the **Funding** statement. The grant number was incorrect. The correct **Funding** statement appears below:

“This study is supported by the National Key R&D Program of China (2018YFC1315200)”.

The authors apologize for these errors and state that this does not change the scientific conclusions of the article in any way. The original article has been updated.

